# Supplementary material for: Ecological determinants of Cope’s rule and its inverse
Source: Commun Biol. 2024 Jan 18;7:38. doi: 10.1038/s42003-023-05375-z (PMC10796397; doi:10.1038/s42003-023-05375-z)
Supplement: Supplementary file 2 — Description of Additional Supplementary Files [file 42003_2023_5375_MOESM2_ESM.pdf]

Description of Additional Supplementary Files

File name: Movie S1
Description: Dynamics of the community coevolution shown in Figure 1.

File name: Movie S2
Description: Dynamics of the community coevolution shown in Figure 2.

File name: Movie S3
Description: Dynamics of the community coevolution shown in Figure 3.

File name: Movie S4
Description: Dynamics of the community coevolution shown in Figure 4.
